# Development and validation of nomogram to predict risk of survival in patients with laryngeal squamous cell carcinoma

**DOI:** 10.1042/BSR20200228

**Published:** 2020-08-17

**Authors:** Jie Cui, Liping Wang, Waisheng Zhong, Zhen Chen, Xiaojun Tan, Hong Yang, Jie Chen, Genglong Liu

**Affiliations:** 1 Department of Head and Neck Surgery, Affiliated Cancer Hospital and Institute of Guangzhou Medical University, Guangzhou 510095, Guangdong Province, PR China; 2 Department of Otorhinolaryngology Head and Neck Surgery, The First Affiliated Hospital of Hainan Medical University, Haikou 570102, Hainan Province, PR China; 3 Department of Head Neck Surgery, Hunan Cancer Hospital and The Affiliated Cancer Hospital of Xiangya School of Medicine, Central South University, Changsha 410000, Hunan Province, PR China; 4 Department of Intensive Care Unit, Shunde Hospital, Southern Medical University (The First People’s Hospital of Shunde), Foshan 528308, Guangdong Province, PR China; 5 Department of Pathology, Affiliated Cancer Hospital and Institute of Guangzhou Medical University, Guangzhou 510095, Guangdong Province, PR China

**Keywords:** laryngeal squamous cell carcinoma, overall survival, nomogram, prediction, surgical treatment

## Abstract

To the best of our knowledge, this is the first study established a nomogram to predict survival probability in Asian patients with LSCC. A risk prediction nomogram for patients with LSCC, incorporating easily assessable clinicopathologic factors, generates more precise estimations of the survival probability when compared TNM stage alone, but still need additional data before being used in clinical application.

**Background:** Due to a wide variation of tumor behavior, prediction of survival in laryngeal squamous cell carcinoma (LSCC) patients received curative-intent surgery is an important but formidable challenge. We attempted to establish a nomogram to precisely predict survival probability in LSCC patients.

**Methods:** A total of 369 consecutive LSCC patients underwent curative resection between 2008 and 2012 at Hunan Province Cancer Hospital were included in the present study. Subsequently, 369 LSCC patients were assigned to a training set (*N*=261) and a validation set (*N*=108) at random. On the basis of multivariable Cox regression analysis results, we developed a nomogram. The predictive accuracy and discriminative ability of the nomogram were confirmed by calibration curve and a concordance index (C-index), and compared with TNM stage system by C-index, receiver operating characteristic (ROC) analysis.

**Results:** Six independent parameters to predict prognosis were age, pack years, N-stage, lymph node ratio (LNR), anemia and albumin, which were all assembled into the nomogram. The calibration curve verified excellent models’ concordance. The C-index of the nomogram was 0.73 (0.68–0.78), and the area under curve (AUC) of nomogram in predicting overall survival (OS) was 0.766, which were significantly higher than traditional TNM stage. Decision curve analysis further demonstrated that our nomogram had a larger net benefit than the TNM stage.

**Conclusion:** A risk prediction nomogram for patients with LSCC, incorporating easily assessable clinicopathologic factors, generates more precise estimations of the survival probability when compared TNM stage alone, but still need additional data before being used in clinical application.

## Introduction

Laryngeal squamous cell carcinoma (LSCC) is among the most frequently diagnosed head and neck squamous cell cancers (HNSCC), with almost 26,300 new cases and about 14,500 deaths in China in 2015 [1]. According to reports, the 5-year overall survival rate varies between approximately 50% and 60%, depending on treatment model, tumor-related factors, and patient-related factors [2]. A variety of treatments are applied to cure LSCC patients, including surgery, radiotherapy and chemotherapy [3]. Despite advances in therapies, the 5-year survival rate was still on the decline according to the review of American Cancer Society [4,5]. On account of a broad spectrum of tumor histological subtypes and diverse clinical behaviors, the prediction of survival risk in patients with LSCC is a difficult task for clinicians. Therefore, it is of great importance to identify reliable and convenient predictive parameters/models to predict survival and optimize therapeutic strategies in LSCC patients.

A mass of predictive variables have been found in LSCC patients [6,7]. Unfortunately, due to the small number of patients, heterogeneous histological subtype and treatment modalities, the impact of clinical factors on survival risk was inconsistent and varies widely. Currently, the tumor-node-metastasis (TNM) stage [8], based on anatomical information, is a common tool to evaluate the prognosis of patients. Though the TNM stage works well on the population level, it less effectively prognosticates at an individual level. Several nomograms, visual description of predictive statistical models for personalized patients, have been built to predict the survival probability in patients with laryngocarcinoma [9–13]. But these studies enrolled different patient cohorts (e.g., laryngeal cancer, advanced laryngeal cancer, and distant metastatic laryngeal carcinoma), and treatment modalities (i.e., radiotherapy vs. surgery). Moreover, all these prediction models were created based on the population of western countries [9–13]. Due to difference in environmental exposures, ethnical diversity [14], adjuvant treatment after operation and treatment modalities, the clinical features of LSCC in eastern countries are also different from western countries. Such discrepancies may lead to inaccurate prediction of clinical outcome if using the same model for the eastern population. For example, the infection rate of human papillomavirus (HPV) is observably different between westerner and easterner in laryngeal carcinoma [15], and HPV positive patients were sensitive to radiotherapy and chemotherapy and showed superior survival [16,17]. Furthermore, the difference of demographic and socio-economic status (SES) may also affect survival outcome of LSCC patients [18].

In the present study, we attempted to develop and internally validate a nomogram to aid decision making in Asian patients with LSCC after surgical resection. Additionally, we compared prediction performance and clinical usability between the nomogram and TNM stage. Notably, the nomogram will allow for tailoring of the therapy to personalized patients in the long term (e.g., for the choice adjuvant treatment).

## Methods

### Patients

Patients with LSCC who after radical surgery were consecutively recorded between October 2008 and November 2012 at Hunan Province Cancer Hospital, with follow up until March 2018. The inclusion criteria were as follows: (a) patients were older than 18 years old; (b) patients with LSCC diagnosed by multidisciplinary teams, including clinicians, radiologist and pathologist; (c) without neoadjuvant chemotherapy or radiotherapy before underwent laryngectomy. The exclusion criteria were as follows: (a) patients with metastasis at diagnosis; (b) patients with other synchronous cancers; (c) indeterminate or incomplete clinicopathologic information; Consequently, a total of 369 patients with LSCC, were enrolled in the present study, diagnosed from October 2008 to November 2012. Subsequently, 369 LSCC patients were assigned to a training set (*N*=261) and a validation set (*N*=108) by R software at random. This retrospective study was endorsed by the ethics committee of Hunan Province Cancer Hospital, which waived the need for informed consent. The trial was registered with ClinicalTrials.gov (NCT03747783).

### Data extraction and follow-up

Clinicopathologic baseline characteristics, including demographics, laboratory tests and pathologic reports were retrospectively extracted for each patient from the HIS medical system of our hospital. According to American Joint Committee on Cancer (AJCC) tumor-node -metastasis (TNM) stage system eighth edition, the TNM stage of LSCC was affirmed by primarily pathological results and secondly imaging reports [8]. Laboratory analysis of hemoglobin and albumin was done via routine blood detections within 1 week before surgery. Anemia was defined as hemoglobin level below 13.7 g/dl for men and below 12.1 g/dl for woman, which corresponds to 8.5 and 7.5 mmol/l, respectively. The threshold value for albumin level was <3.5 and ≥3.5 g/dl, according to the normal range used at our institution. After classifying the patients with survival status, we computed the optimal cutoff points of lymph node ratio (LNR) by receiver operating curve (ROC) analysis according to the maximum Youden index (sensitivity+specificity−1).

The standard operation for LSCC was a total or partial laryngectomy or transoral laser microsurgery determined according to the characteristics of the case (mainly the stage) by the Head and Neck Cancer Committee, with preserving the function of the larynx as much as possible. Curative resection was defined as no tumor remaining after removal, both macroscopically and microscopically. The clinical endpoint was overall survival (OS), defined as the time from surgery to death. In addition, patients who were alive were counted as censored observations at the time of last follow-up. After completion of surgical therapy, patients received regular follow-up from their surgeon and/or the surveillance team every 3 months for the first year, every 3–6 months for the second and third years, and every 6 months thereafter. All living patients who did not show up for a scheduled check-up or who were lost to follow up were reminded by telephone.

### Statistical analysis

Nonnormally distributed data were described as median interquartile range and normally distributed data were presented as mean (standard deviation), which were tested by Wilcoxon rank sum tests or *t* tests following the distribution of the parameters. Categorical variables were reported as number (percentage), which were compared using Chi-square or Fisher’s exact tests.

Given the potential prognostic value of the clinicopathologic variables, univariate Cox regression analysis was applied to preliminarily identify clinical risk parameters related to OS in the training set. Linearity assumption for continuous variables was evaluated by restricted cubic splines [19]. According to clinical reasoning, categorical variables were grouped, which were made before modeling. Variables were subjected to multivariable Cox regression analysis when its *P* value below 0.05 or less in the univariate analyses. Additionally, to investigate the degree of multicollinearity among variables, the variance inflation factor (VIF) was calculated in the multivariable Cox regression analysis. If VIF was >10, then multicollinearity was high [20].

According to the results of multivariable Cox regression analysis, a nomogram was constructed, which selects the significantly relevant OS factors by a backward step-down process with the Akaike information criterion [21]. The performance of the nomogram was evaluated in training and validation sets in terms of discrimination and calibration. The discrimination performance for predicting recurrence was numerically assessed by calculating Harrell’s concordance index (C-index). In addition, according to the height of the linear predictor which is summed up all regression coefficients from an individual patient, we investigated discrimination ability by dividing the dataset into four groups. Discrimination was visualized by plotting a Kaplan–Meier curve with Log-rank test for all four groups. The calibration curve was plotted to evaluate the degree of fitting of the nomogram.

Furthermore, receiver operating characteristic (ROC) curves analysis was carried out to estimate and compare the discrimination ability of the nomogram to TNM stage for predicting 3- and 5-year RFS with area under curve (AUC) value. To evaluate the clinical usability of the nomogram, decision curve analysis (DCA), as a comprehensive method for estimating and comparing between nomogram and TNM stage, was conducted by computing the net benefits for a range of threshold probabilities [22].

The VIFs were done by “car” package. The ROC curves were plotted by “surivivalROC” package. Nomogram establishment and calibration plots were conducted using the “rms” package. DCA was performed using the “stdca.R”. SPSS statistics 22.0 and R software (R version 3.5.2) were used to perform the statistical analysis. A two-sided ***P*** value of 0.05 or less was considered significant.

## Results

### Basic clinicopathologic characteristics

Table 1 displayed the characteristics of 261 LSCC patients in the training cohort and 108 patients in the validation cohort. The median follow-up time was 46 months (range = 2–67 months) and 45 months (range = 2–67 months) for training set and validation set, respectively. Of all the 369 LSCC patients, 249 patients (67.5%) survived during follow-up. The estimated 3- and 5-year OS rates were 74% (70–78.1%) and 63.5% (58.8–68.2%) in the training set, respectively. Similarly, the estimated 3- and 5-year OS rates were 73.9% (67.4–80.4%) and 67.7% (60.8–74.6%) in the validation set, respectively. Supplementary Table S1 summarized a detailed analysis of both sets.

**Table 1:** Characteristics of patient in the training set and validation set

Variable	Category	Training set		Validation set	
		(*n*=261)	%	(*n*=108)	%
**Age**	Median (years)	60		59	
	Range (years)	36–80		36–77	
**Sex**	Male	250	95.8	99	91.7
**Tumor site**	Supraglottic	94	36	37	34.3
	Glottic	161	61.7	67	62
	Subglottic	6	2.3	4	3.7
**Smoking**	Yes	159	60.9	71	65.7
**Mean pack years**	Median	20		20	
	Range	0–50		0–50	
**Alcohol**	Yes	78	29.9	32	29.6
**T-stage**	T1	27	10.3	6	5.6
	T2	102	39.1	45	41.7
	T3	105	40.2	49	45.4
	T4a	27	10.3	8	7.4
**N-stage**	N0	157	60.2	66	61.1
	N1	58	22.2	30	27.8
	N2	46	17.2	12	11.1
	N3	1	0.4	0	0
**Number of LN**	Median	9		12	
	Range	0–58		0–58	
**Number of positive LN**	Median	0		0	
	Range	0–28		0–14	
**LNR**	**<**0.13 g/dl	205	78.5	78	72.2
	≥0.13 g/dl	56	21.5	30	27.8
**TNM stage**	Stage I	21	8.8	5	4.6
	Stage II	78	29.9	33	30.6
	Stage III	101	38.7	50	46.3
	Stage IVa	61	23.4	20	18.5
**Histology grade**	Moderate-Well	249	95.4	103	95.4
	Poor	12	4.6	5	4.6
**Anemia**	Yes	69	26.4	27	25
**Albumin**	**<**3.5 g/dl	45	17.2	20	18.5
	≥3.5 g/dl	216	82.8	88	81.5

Abbreviations: LN, lymph node; LNR, lymph node ratio; TNM, tumor node metastasis.

### Identification of OS relevant factors

Based on univariate analysis in the training set, we appraised eight parameters, including age, pack years, N-stage, number of positive of lymph node (PLNs), lymph node ratio (LNR), TNM stage, anemia and albumin, were associated with OS probability (Table 2). Multivariable analyses continued to confirm that age, pack years, N-stage, lymph node ratio (LNR), anemia and albumin were independent risk factors for OS. In terms of the collinearity diagnosis, the VIFs of the six predictors varied between 1.02 and 2.58, confirming that there was no collinearity.

**Table 2:** Univariable and multivariable Cox regression analysis for the prediction of survival

Factors	Subgroup	Univariable analysis		Multivariable analysis	
		HR (95%CI)	*P*	HR (95%CI)	*P*
**Age**		1.04(1.02–1.07)	0.002*	1.03 (1.01–1.06)	0.021*
**Sex**	Female	1			
	Male	4.49 (0.63–32.3)	0.135	NA	NA
**Tumor site**	Glottic	1			
	Supraglottic	1.53 (0.99–2.37)	0.057	NA	NA
	Subglottic	1.68 (0.52–5.41)	0.384	NA	NA
**Smoking**	No	1		1	
	Yes	1.54 (0.98–2.44)	0.063	1.37 (0.49–3.82)	0.549
**Pack years**		1.02 (1.01–1.03)	0.008*	1.02 (1.01–1.03)	0.017*
**Alcohol**	No	1			
	Yes	1.01 (0.64–1.61)	0.962	NA	NA
**T-stage**	T1	1			
	T2	1.32 (0.58–3.0)	0.506	NA	NA
	T3	1.73 (0.77–3.88)	0.181	NA	NA
	T4a	1.86 (0.68–5.14)	0.229	NA	NA
**N-stage**	N0	1	1		
	N1	2.84 (1.72–4.71)	0.000*	1.95 (1.08–3.52)	0.028*
	N2-3	3.02 (1.77–5.16)	0.000*	1.54 (1.01–2.25)	0.048
**Number of LN**		1.0 (0.98–1.03)	0.726	NA	NA
**PLN**		1.06 (1.01–1.12)	0.023*	0.97 (0.88–1.07)	0.576
**LNR**	**<**0.14 g/dl	1		1	
	≥0.14 g/dl	2.58 (1.68–4.07)	0.001*	2.26 (1.42–3.60)	0.001*
**TNM stage**	Stage I	1			
	Stage II	0.96 (0.35–2.60)	0.936	NA	NA
	Stage III	2.09 (1.03–5.33)	0.042*	1.59 (0.82–4.26)	0.122
	Stage IVa	2.50 (1.26–6.51)	0.014*	1.80 (0.96–5.51)	0.101
**Histology grade**	Moderate-Well	1			
	Poor	1.59 (0.69–3.64)	0.276	NA	NA
**Anemia**	No	1		1	
	Yes	1.82 (1.12–2.87)	0.008*	1.81 (1.15–2.88)	0.011*
**Albumin**	≥3.5 g/dl	1		1	
	<3.5 g/dl	1.68 (1.02–2.78)	0.042*	1.69 (1.01–2.83)	0.046*

Abbreviations: CI, confidence intervals; HR, hazard ratio; LN, lymph node; LNR, lymph node ratio; PLN, number of positive LN.

NOTE: NA, not available. These variables were eliminated in the multivariate cox regression model, so the HR and ***P*** values were not available.

* ***P***<0.05.

### Development and validation of the nomogram

**Figure 1:**
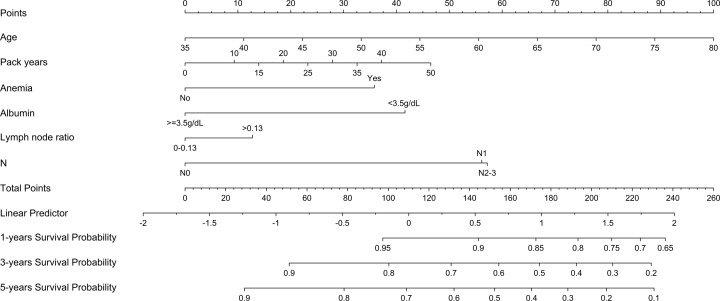
Nomogram for predicting 1-year, 3-year and 5-year survival probability of LSCC after laryngectomy To estimate risk, calculate points for each variable by drawing a straight line from patient's variable value to the axis labeled “Points". Sum all points and draw a straight line from the total point axis to the 1-, 3- and 5-year survival axis.

**Figure 2:**
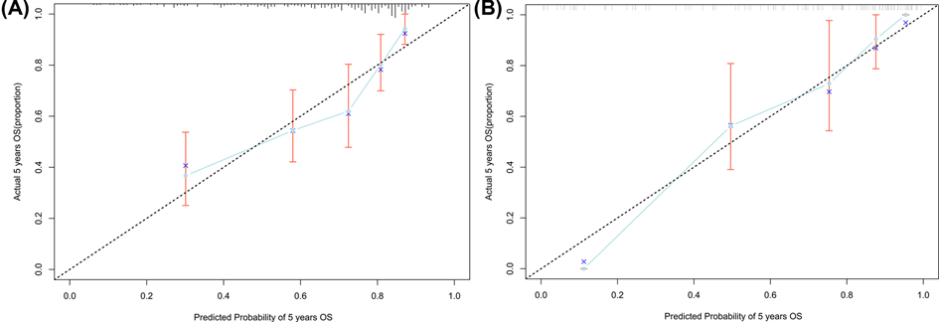
Calibration curves for nomogram Calibration curves for (**A**) 5-year nomogram in the training set, and (**B**) 5-year nomogram in the validation set. Patients were grouped by octiles of predicted risk. *X*-axis is nomogram-predicted probability of survival(LSCC). *Y*-axis is observed probability of LSCC (Kaplan–Meier estimates). Broken line = ideal nomogram; circles = apparent predictive accuracy, calculated by plotting the mean Kaplan–Meier estimate for each octile versus the mean nomogram-predicted probabilities for patients in each octile; X's = bootstrap-corrected estimates; vertical bars = 95% CIs.

**Figure 3:**
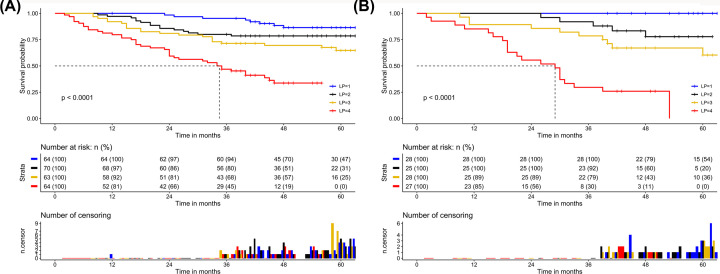
Kaplan–Meier curves of four groups Kaplan–Meier curves of four groups based on the linear predictor (**A**) in the training set, and (**B**) in the validation set.

According to the results of multivariable Cox regression analysis, we built a nomogram to predict 1-, 3- and 5-year OS in the training set (Figure 1). The C-index of the nomogram for OS prediction was 0.73 (0.68–0.78) (Table 3), and in Figure 2A and Supplementary Figure S1A, the calibration plot represents that predicted survival is corresponding with actual survival. Likewise, consistent findings were also presented in the validation set. The C-index of the nomogram for OS prediction was 0.84 (0.78–0.89) (Table 3) and also presented good consistency between the predicted OS and the actual OS (Figure 2B and Supplementary Figure S1B). Additionally, based on the linear predictor, we divided the data into four groups. The Kaplan–Meier analysis (Log-rank *P*<0.0001) of the four risk subgroups indicated the great discrimination of the nomogram in training set (Figure 3A) and in validation set (Figure 3B).

**Table 3:** Assessing the prognostic performance of the TNM stage and nomogram in training set and validation set

Cohort	Model	Homogeneity monotonicity and discriminatory ability			
		Likelihood ratio (LR) test*	Linear trend χ2 test^†^	C-index (95% CI) ^‡^	Akaike information criterion (AIC)^§^
Training set	TNM stage	10.3	9.6	0.59 (0.54–0.65)	892.4
	Nomogram	56.4	62.1	0.73 (0.68–0.78)	856.3
Validation set	TNM stage	1.8	1.8	0.57 (0.49–0.66)	320.3
	Nomogram	58.3	61	0.84 (0.78–0.89)	275.5

* Higher homogeneity likelihood ratio indicates a smaller difference within the staging system, it means better homogeneity.

† Higher discriminatory ability linear trend indicates a higher linear trend between staging system, it means better discriminatory ability and gradient monotonicity.

‡ A higher c-index means better discriminatory ability.

§ Smaller AIC values indicate better optimistic prognostic stratification.

### Comparison of predictive accuracy and clinical usefulness between nomogram and TNM stage

**Figure 4:**
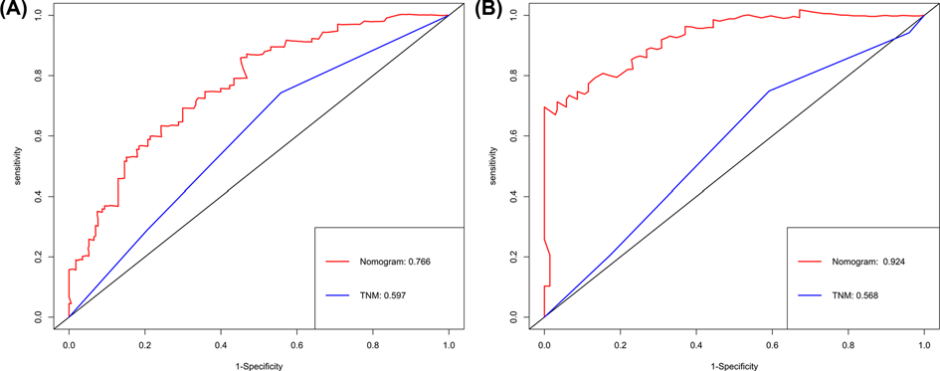
ROC curves compare the prediction accuracy of the nomogram with TNM stage ROC curves compare the prediction accuracy of the nomogram with TNM stage in predicting 5-year OS (**A**) in the training set, and (**B**) in the validation set.

**Figure 5:**
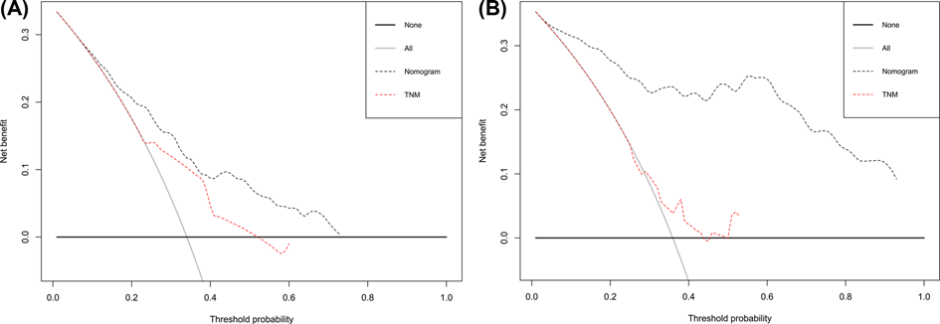
Decision curve analysis for the nomogram and TNM stage Decision curve analysis for the nomogram and TNM stage in prediction of prognosis of patients at 5-year point (**A**) in the training set, and (**B**) in the validation set.

To further evaluate the predictive ability of nomogram, we compared nomogram with TNM stage model in both set. As was shown at Table 3, the C-index of nomogram was better than that of TNM stage, 0.59 (0.54–0.65) in the training set and 0.57 (0.49–0.66) in the validation set. Likelihood ratio test, linear trend χ2 test and Akaike information criterion all demonstrated that the nomogram had better prediction efficiency than the TNM stage alone. ROC analysis also indicated that the nomogram was better than TNM stage alone (3-year AUC 0.762 vs 0.619 for the training set, 3-year AUC 0.902 vs 0.582 for the validation set and 5-year AUC 0.766 vs 0.597 for the training set, 5-year AUC 0.924 vs 0.568 for the validation set) in predicting 3-year OS (Supplementary Figure S2A and S2B) and 5-year OS (Figure 4A,B). Finally, DCA was applied to compare the clinical usefulness of the nomogram to that of traditional TNM stage. DCA graphically showed that the nomogram was better than traditional TNM stage in both set in predicting 3 years OS (Supplementary Figure S3A and S3B) and 5 years OS (Figure 5A,B).

## Discussion

Using a multivariate Cox regression analysis for a number of readily available clinicopathologic variables in a large group of unselected patients with LSCC, we developed a visual, ready-to-use nomogram for LSCC patients to predict survival outcome following curative resection. The nomogram accurately predicted survival probability, with a bootstrapped corrected C-index of 0.73, and 0.84 with an internal validation cohort. Additionally, the nomogram showed better predictive ability and clinical usability than TNM stage for predicting the survival of LSCC patients.

A number of risk-prediction nomograms have been published in recent years.

Egelmeer et al. [9] conducted the initial nomogram using a study cohort of 994 patients with laryngeal cancer receiving radiotherapy alone from 1977 to 2008 in the Netherlands. Consistent with our findings, they revealed that older age, hemoglobin level and higher *N* status to be poor prognosis for OS. The model might produce pessimistic survival estimates on account of continual refinement and evolution of treatment over the past four decades. In 2017, Multidisciplinary Larynx Cancer Working Group [10], using data from University of Texas MD Anderson Cancer Center database, constructed a dynamic risk model and clinical nomogram in locally advanced laryngeal patients by conditional survival analysis. They uncovered age and nodal burden to be significant for 3- or 6-year OS in the multivariate analysis, in compliance with our results. As the authors point out, the model might not be generalizable due to the absence of external validation. Another risk prediction model based on 2752 LSCC patients underwent neck dissection in the Surveillance, Epidemiology, and End Results (SEER) database between 1988 and 2008 has been developed by Shi et al. [11]. The nomogram was constructed following eight independent prognostic clinical variables. However, only 20 patients were in an undifferentiated subset, which probably reduced the accuracy of the prediction. Sequentially, Hoban et al. [12] established a prognostic model for patients with laryngeal cancer. The analysis data set was collected in University of Michigan Health System, which included 246 cases confirmed by biopsy, TNM stage I to IVb, previously untreated with laryngeal squamous cell carcinoma between 2003 and 2014. Missing variables were a source of imperfection in this estimation, which lead to potentially biassed results and a loss of statistical power [23]. Recently, Petersen et al. study [13], which mined data covering patients diagnosed with advanced LSCC in the Netherlands (1991–2010), developed and validated a nomogram to predict 5-year OS in advanced SCC of the larynx. The study provides a C-index of 0.65, and 0.59 with an external validation set. The accuracy of this nomogram may have been limited by its neglect of several important clinical and laboratory prognostic indices, such as smoking status, hemoglobin value, and albumin level that are not available in the database.

To the best of our knowledge, this is the first study established a nomogram to predict survival probability in Asian patients with LSCC. We identified six independent predictors that age, pack years, N-stage, LNR, anemia and albumin, were extraordinarily frequent reported prognostic predictors in patients with LSCC [9–13,24–27]. In the present study, in terms of risk stratification of the model, ability of discrimination and homogeneity, the performance of the nomogram in predicting survival ability is superior to the TNM staging. The strength of the current nomogram is that it incorporated clinicopathological factors, which are critical of importance for predicting recurrence risk, but cannot be adopted by TNM stage system. Remarkably, DCA results showed that LSCC survival-related treatment decisions according to the nomogram led to more net benefit than treatment decisions based on TNM stage, or treating either all patients or none. Taken together, the present nomogram would be clinically useful for the clinicians in tailoring survival-associated treatment decisions.

Although our nomogram demonstrated impressive performance in LSCC survival prediction, there are specific limitations associated with our trial. First, the study is a typical retrospective analysis conducted in a single institution. Because the database derived from a single tertiary cancer center, the model may only represent a kind of patient sample that cannot be extended to the general population. In spite of perfect internal validation, the presented nomogram was not yet suitable for wide use prior to validation of external cohorts. Thus, external and multicenter prospective cohorts with large sample sizes are still needed to validate the clinical application of our model.

Second, the present study is an observational cohort trial, which enrolled LSCC patients who underwent laryngectomy alone at our institution. Given treatment decision was made before inclusion in the trial, there is a potential selection bias. Moreover, our nomogram was not applied to predict survival in LSCC patients with other radical treatment models, including radiotherapy and chemotherapy.

Third, the factors we choose were limited to those available in our database. Considering the database was confined, we cannot extend our variables. To begin with, comorbidity scores can be of great importance in survival predictions. Nevertheless, comorbidity scores were of absence in our cohort. In 2013, a risk-prediction nomogram for head and neck cancer incorporated the Adult Comorbidity Evaluation-27 (ACE-27) as a prognostic factor [28]. The effect of severe comorbidity seems to be comparable to that of T4 tumors or N3 on overall survival in their models. Another nomogram with exploratory post hoc analysis, adding American Society of Anesthesiologists (ASA) score which served as a replacement measure for comorbidity scores as a prognostic factor, increased C-index from 0.65 to 0.68 in advanced larynx cancer [13]. But we were not able to integrate the impact of comorbidity on nomogram which may limit its performance of prediction. Though comorbidity would certainly affect overall survival estimates, our nomogram yielded similar C-index in the validation datasets and was much better than TNM stage. Next, we did not take into consideration socio-economic status (SES). A number of studies have proven the value of SES in HNSCC [29,30]. Hence, we recommend that future studies should investigate whether SES might be value prediction to survival outcome in LSCC, and evaluate the additional worth of this factor in a multivariable prediction model. Final, the nomogram did not consider human papillomavirus (HPV). Published researches and meta-analysis indicated HPV positivity in patients with LSCC could be associated with a favorable prognosis, independently of other clinical-pathological factors [31,32].

Fourth, anemia is considered to be one of the diagnostic standard for tumor cachexia, accompanied by weight loss. As we know, tumor cachexia has a negative effect on OS, so it may be a potential confounding factor [33]. Hence, the role of anemia and cancer cachexia as independent predictors of survival needs further research.

Fifth, with the advent of modern gene-array and immunohistochemistry technology, molecular variables may help predict survival probability of LSCC. As we know, LSCC is a heterogeneous disease, which means that it needed better tools to assist patients and physicians to make choices in treatment options. The combination of clinical, pathologic and molecular variables may be finally uncover more robust and powerful measures for stratification of disease risk than any single approach.

## Conclusion

We have built visual, easy-to-use nomogram, based on a large system coding database, for the prediction of survival and with several easily available clinicopathologic variables in patients with LSCC. The predictive ability and clinical usefulness of nomogram are significantly better than of the TNM stage alone. Although it is not a substitute for clinical reasoning, it may be helpful to the decision-making process of LSCC patients.

## Supplementary Material

Supplementary Figures S1-S3 and Table S1

## Data Availability

The dataset analyzed for the current study are available from the corresponding author on reasonable request.

## Abbreviations

ACE-27: Adult Comorbidity Evaluation-27
AJCC: American Joint Committee on Cancer
ASA: American Society of Anesthesiologists
AUC: area under curve
DCA: decision curve analysis
HNC: head and neck cancer
HNSCC: head and neck squamous cell cancers
HPV: human papillomavirus
LNR: lymph node ratio
LSCC: laryngeal squamous cell carcinoma
OS: overall survival
ROC: receiver operating characteristic
SEER: Surveillance, Epidemiology, and End Results
SES: socio-economic status
TNM: tumor-node-metastasis
VIF: variance inflation factor
