# Supplementary material for: Development and validation of nomogram to predict risk of survival in patients with laryngeal squamous cell carcinoma
Source: Biosci Rep. 2020 Aug 17;40(8):BSR20200228. doi: 10.1042/BSR20200228 (PMC7432998; doi:10.1042/BSR20200228)
Supplement: Supplementary Figures S1-S3 and Table S1 [file BSR-2020-0228_supp.pdf]

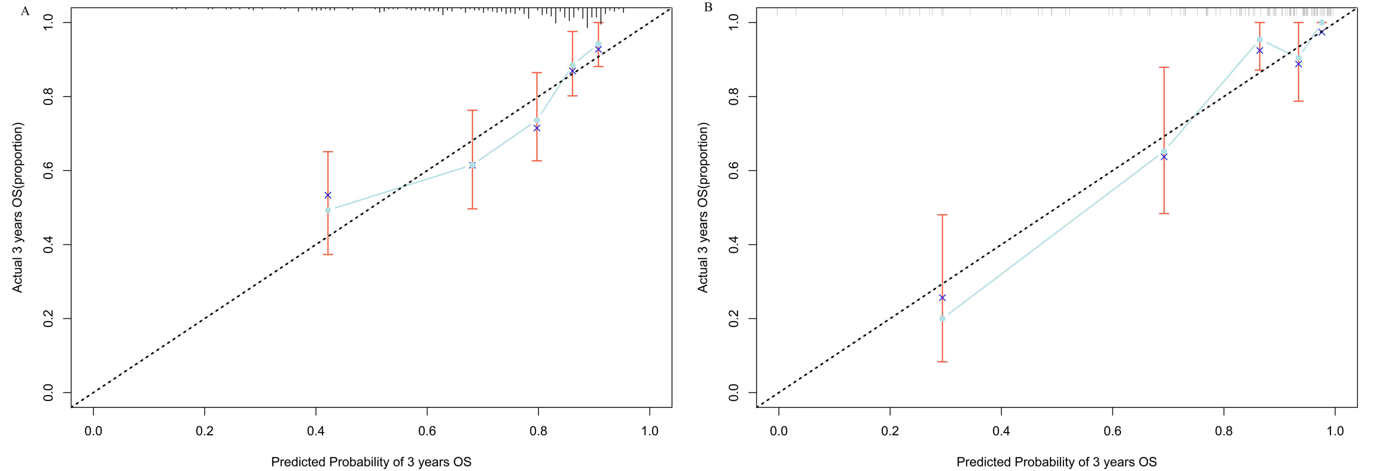

A
B
Actual 3 years OS(proportion)
Predicted Probability of 3 years OS
0.0
0.2
0.4
0.6
0.8
1.0

FigureS1. Calibration curvesfor (A) 3-year nomogram in the training set, and (B) 3-year nomogram in the validation set. Patients were grouped by octiles of predicted risk. x-axis is nomogram-predictedprobability of survival(LSCC). y-axis isobserved probability of LSCC (Kaplan-Meierestimates). Broken line = ideal nomogram;circles = apparent predictive accuracy, calculated by plotting the mean Kaplan-Meierestimate for each octile versus the meannomogram-predicted probabilities for patients in each octile; X’s = bootstrap-correctedestimates; vertical bars = 95% CIs.

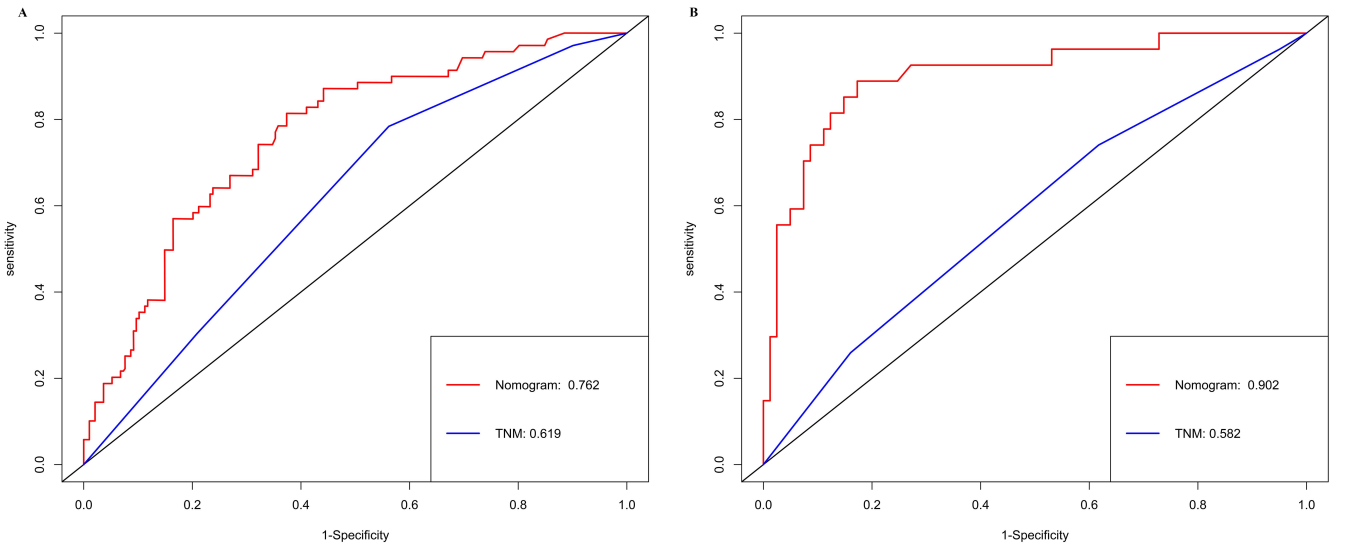
A
B
sensitivity
1-Specificity
0.0
0.2
0.4
0.6
0.8
1.0
Nomogram: 0.762
TNM: 0.619
Nomogram: 0.902
TNM: 0.582

Figure S2. ROC curves compare the prediction accuracy of the nomogram with TNM stagein predicting 3-year OS (A) in the training set, and (B) in the validation set.

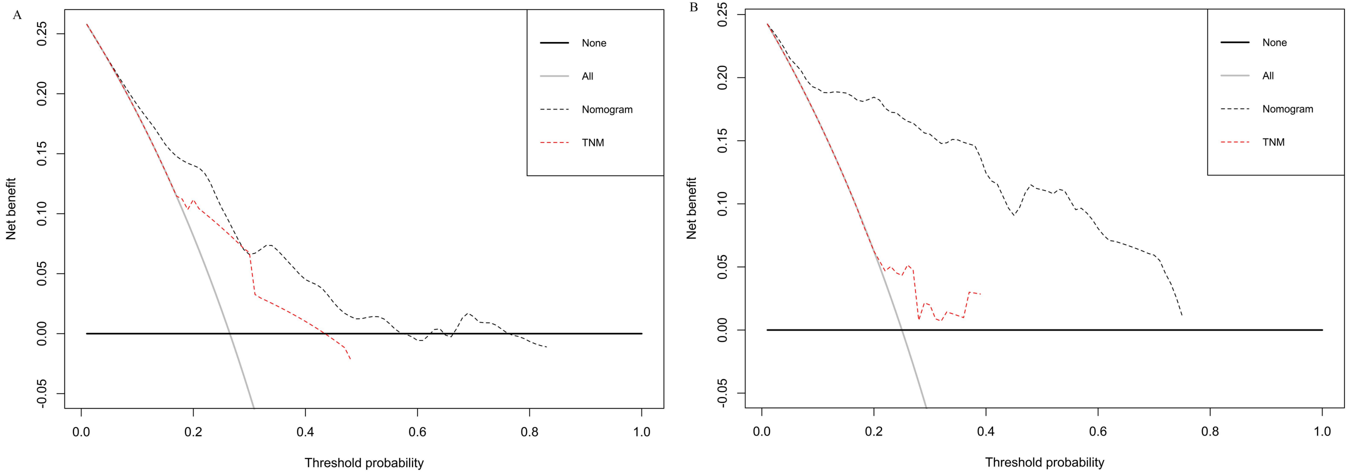
A
B
None
All
Nomogram
TNM
Net benefit
Threshold probability
0.25
0.20
0.15
0.10
0.05
0.00
-0.05
0.0
0.2
0.4
0.6
0.8
1.0

Figure S3. Decision curve analysis for the nomogram and TNM stage in prediction of prognosis of patients at 3-year point (A) in the training set, and (B) in the validation set.

TableS1 summarized a detailed analysis of both sets**.**

Means and Medians for Survival Time				
**Group**			**Mean[a]**	
	**Estimate**	**Std. Error**	**95% Confidence Interval**	
			**Lower Bound**	**Upper Bound**
**Training set**	68.887	1.537	65.874	71.900
**Validation set**	54.977	1.569	51.903	58.051
**Overall**	69.263	1.287	66.740	71.786

	Survival probability			
	**No**	**1-year**	**3-year**	**5-year**
**Training set**	431	96.3(94.5-98)	74(70-78.1)	63.5(58.8-68.2)
**Validation set**	180	93.3(89.6-97)	73.9(67.4-80.4)	67.7(60.8-74.6)

	Follow up time			
	**Median(months)**	**Range(months)**	**1Qs(months)**	**3Qs(months)**
**Training set**	46	2-67	33.5	60
**Validation set**	45	2-67	34.75	60

Qs=Quadrangle spacing
